# Interoception, alexithymia, and anxiety among individuals with alcohol use disorder

**DOI:** 10.3389/fpsyt.2023.1229985

**Published:** 2023-09-22

**Authors:** Paweł Wiśniewski, Andrzej Jakubczyk, Elisa M. Trucco, Paweł Kobyliński, Hubert Suszek, Justyna Zaorska, Małgorzata Rydzewska, Maciej Kopera

**Affiliations:** ^1^Department of Psychiatry, Medical University of Warsaw, Warsaw, Poland; ^2^Department of Psychology, Center for Children and Families, Florida International University, Miami, FL, United States; ^3^Department of Psychiatry, Addiction Center, University of Michigan, Ann Arbor, MI, United States; ^4^Laboratory of Interactive Technologies, National Information Processing Institute, Warsaw, Poland; ^5^Faculty of Psychology, University of Warsaw, Warsaw, Poland

**Keywords:** interoception, interoceptive accuracy, alexithymia, negative affect, alcohol use disorder

## Abstract

**Background:**

Interoception (i.e., the ability to recognize bodily signals), alexithymia (i.e., the inability to recognize emotional states) and negative affect (i.e., unpleasant feelings such as anxiety) have been associated with alcohol use disorder (AUD). Previous research suggests that interoception may underlie alexithymia, which in turn may be associated with negative affectivity. However, this remains to be empirically tested. This study investigates whether alexithymia mediates the association between interoception and anxiety and whether this association differs across individuals with AUD and a healthy control (HC) comparison group.

**Methods:**

The AUD group consisted of 99 participants enrolled in an 8-week abstinence-based inpatient treatment program. The HC group included 103 healthy individuals. The heartbeat counting task (HCT) was used to assess interoception (cardiac interoceptive accuracy). The Toronto Alexithymia Scale (TAS-20) was used to assess alexithymia. The Brief Symptom Inventory (BSI) was used to assess anxiety.

**Results:**

The moderated mediation model with interoception as the predictor, alexithymia as the mediator, and negative affect (i.e., state anxiety) as the dependent variable was tested. The analysis showed that the conditional indirect effect of interoception on anxiety via alexithymia was significant for individuals with AUD [*ab* = −0.300, bootstrap 95% CI = (−0.618, −0.088)], as well as for HCs [*ab* = −0.088, bootstrap 95% CI = (−0.195, −0.014)]; however, the conditional indirect effect significantly differed across HCs and individuals with AUD. Namely, the mediated effect was greater among individuals with AUD compared to the HC group.

**Conclusion:**

The results suggests that interoceptive impairment contributes to greater negative affect (i.e., state anxiety) via alexithymia especially for individuals with AUD. Improving emotion recognition via therapeutic methods focused on strengthening interoceptive abilities could improve outcomes for individuals receiving treatment for AUD.

## Introduction

Negative affect (i.e., a state of emotional distress associated with unpleasant feelings, such as anxiety, fear, irritability, and sadness) has been shown to be one of the most crucial factors in the development and course of substance use disorder (SUD). Its role in addiction is particularly important according to various theories of negative reinforcement (1–3). These theories emphasize the role that substances play in reinforcing use based on how emotions are experienced. For example, Koob’s model of allostatic dysregulation depicts addiction as a multistage process that involves both positive (e.g., pleasant feelings that the substance elicits) and negative (e.g., reducing unpleasant feelings) reinforcement mechanisms (4). Moreover, this model conceptualizes addiction as a disorder that progresses from positive reinforcement to negative reinforcement. According to Hogarth, the negative reinforcement mechanism may be even more relevant to addiction than other mechanisms (such as habit or compulsion) (5). This model indicates that “addiction is primarily driven by an excessive goal-directed drug choice under negative affect.” The negative reinforcement theory also applies to individuals with AUD. For example, referring to classical theories of alcohol consumption, which assume that people use alcohol to cope with negative feelings, Wolkowicz and colleagues showed that *the stress-dampening model* may be more important in the early stages of addiction development, while *the tension-reduction model* may be more relevant in heavy-drinking individuals (e.g., individuals with AUD) (6). Among the various phenomena that support negative affectivity as a catalyst for substance use, anxiety is often indicated as a strong motivator for alcohol use (7). Additionally, anxiety is associated with many physical symptoms that involve bodily sensations. Therefore, in this work, we use state anxiety severity as a measure of negative affect.

Negative affectivity among individuals with AUD may be related to impaired mechanisms of emotion regulation in this group. Koob and Volkow highlight alexithymia as one of the key motivational elements that may be a source of unpleasant feelings that uniquely contributes to the cycle of addiction along with dysphoria, irritability and other factors (8). Alexithymia is a clinical construct defined as an inability to recognize and describe emotional states (9). Indeed, it is associated with negative affect among individuals with AUD (10, 11). Prior work supported the use of alcohol as a maladaptive strategy to cope with negative affective states among individuals high in alexithymia (12). Additionally, alexithymia was associated with an earlier age of alcohol use onset, longer duration of problematic drinking, and greater alcohol consumption (13). Moreover, alexithymia also predicted poorer outcomes among individuals with AUD (14). Although the prevalence rate of alexithymia among individuals with AUD is high with estimates between 30 to 67% (15, 16), the factors underlying this overlap remain unclear. In a recent review, Cruise and Becerra (16) conclude that there is convincing evidence supporting alexithymia as an independent risk factor for alcohol-related problems. Further, they point to the growing evidence indicating that alexithymia may be a mechanism through which alcohol-related problems (e.g., emotion dysregulation) lead to AUD. The authors emphasize that it is clinically important to empirically test the nature of the indirect associations between alexithymia and negative affect to inform intervention programming for AUD (16). Importantly, theories are emerging indicating that alexithymia may be related to interoceptive abnormalities (17, 18). Some of these theories even refer to alexithymia as a “general deficit of interoception” (17).

Interoception is the process of bidirectional communication between the brain and internal organs by sensing and interpreting signals arising from within the body and associating them with external stimuli and memory representations to maintain homeostasis (19, 20). Initially, this process was mainly related to biological aspects, but recently its importance in psychological phenomena (e.g., emotion regulation, cognition, self-awareness) has been emphasized (21, 22). Current research recognizes three key domains of interoception: (1) behaviorally measured accuracy or sensitivity, (2) self-reported sensibility, and (3) metacognitive awareness (23). Impairment in interoceptive abilities has been documented in several psychiatric disorders, such as anxiety, depression, autism, and eating disorders (24, 25). There is also significant literature linking disruptions in interoception with addiction [e.g., (26)]. Namely, the notion of embodiment posits that one’s emotional state when first experiencing the effects of a drug may exacerbate the difference between the predicted and actual internal state of an individual in the future and, consequently, increase negative affect and promote drug seeking-behavior (27). The association between interoception and AUD is complex [for review see: (28, 29)]. Studies demonstrate decreased interoceptive accuracy in individuals with AUD in comparison to healthy controls (30–32). There is also evidence that interoceptive accuracy is negatively correlated with alcohol craving (30) and difficulties in emotion regulation in individuals with AUD (33).

Deficits in accurately perceiving internal bodily signals may underlie abnormal processing of emotions among individuals with alexithymia. Classical theories of emotion indicate that various emotional states may have their physiological basis in the form of primary changes in the body (34, 35). According to the model of Lane and Schwartz (36), experiencing emotions is a complex process of detailing information, from simple physiological changes in the body to distinguishing individual nuanced emotions. The authors described five levels of emotional experience. The first of these levels, somatic sensation activity, refers to signals coming from the body. At this level, individuals only feel bodily sensations and are unable to describe these sensations in detail. Proper recognition of interoceptive signals is a prerequisite for consciously experiencing, distinguishing, and describing emotions. The authors also emphasize the utility of their theory in understanding alexithymia. In their view, individuals with alexithymia are unable to differentiate between feelings. As a result, individuals high in alexithymia experience arousal (negative affect). This phenomenon may be due to abnormal interoception. More recent work confirms that impaired interoception among individuals with alexithymia may be a source of anxiety (37).

Interestingly, at the neurobiological level, alexithymia is associated with functional impairments in brain regions typically involved in the processing of interoceptive information [e.g., the insula; (38)]. Despite the theoretical basis linking interoception and alexithymia, the results of empirical studies reflecting these associations remain unclear. That is, some studies demonstrate a negative correlation between interoceptive accuracy and alexithymia (18, 39–41), while others show a positive correlation (42, 43) or no correlation (18, 44–46) between the two constructs. In their meta-analysis, Trevisan and colleagues confirmed the association between interoceptive sensibility and alexithymia and showed no association between interoceptive accuracy and alexithymia (47). However, available research showed that interoceptive accuracy negatively correlates with alexithymia scores among individuals with AUD (32). The study of Betka and colleagues on the association between interoception and alexithymia among social drinkers showed that impaired interoceptive abilities may underlie alexithymia and thus contribute to the use of alcohol as a maladaptive coping strategy (48).

Available data suggests that high alexithymia may be associated with negative affectivity. This in turn may promote alcohol use. A possible underlying mechanism linking alexithymia and negative affectivity may be impaired interoception (17, 18, 48). Thus, we believe that the degree to which impairment in sensing bodily signals is related to negative affectivity may depend on the ability to recognize and describe emotional states. Although the association between interoception, alexithymia, and negative affect has been studied individually, to the best of our knowledge there has been no research that has investigated the combined association between all three factors with an AUD sample. Therefore, the aim of the current study was to assess whether alexithymia mediates the association between interoception and negative affect (i.e., state anxiety) and whether differences exist across individuals meeting criteria for AUD and a healthy control (HC) comparison group using moderated mediation modeling. We hypothesized that alexithymia would mediate the association between interoceptive accuracy and state anxiety. We did not formulate specific hypotheses relating to possible differences across the two groups.

## Materials and methods

### Participants

The current data comes from an ongoing study examining the emotional and behavioral functioning of individuals with AUD and a HC comparison sample. The study sample consisted of 99 adults (average years of age = 43.4 ± 10.1) who were admitted to an abstinence-based, drug-free, eight-week, inpatient alcohol treatment program incorporating psychoeducation and cognitive-behavioral therapy (CBT). The AUD group consisted of individuals treated in an inpatient setting with severe symptoms of AUD, but without acute withdrawal symptoms. The average duration of abstinence from alcohol was 49.2 ± 45.1 days prior to study enrollment. Study procedures were performed during the first two weeks after treatment admission.

AUD diagnosis using the International Classification of Diseases and Related Health Problems 10th Revision (49) was obtained by a psychiatrist upon treatment admission and then subsequently confirmed via the MINI International Neuropsychiatric Interview (50). Adults with a history of psychosis, current co-occurring mental health disorders requiring medication, current co-occurring substance use disorder other than nicotine, or a clinically significant cognitive deficit (< 25 on the Mini-Mental State Examination) (51) were not eligible.

HCs included 103 adults (average years of age = 40.4 ± 8.4) that met with a general practitioner for a yearly physical examination or for medical advice [see (52) for additional description of the study sample]. In HCs, study procedures were performed prior to the routine visit to their primary care physician. Adults endorsing harmful alcohol use as assessed via the Alcohol Use Disorders Identification Test [AUDIT; (53)] were not eligible. A large portion of the sample encompasses White men (AUD 87%, HC 76%) consistent with the demography of patients admitted in substance use treatment programs in Poland. When comparing groups on demographic factors, the HC sample was significantly younger [F(1, 200) = 5.05, *p* = 0.03] and more likely to be female [χ^2^ (1, 202) = 4.1, *p* = 0.04] compared to the AUD sample. Accordingly, age and biological sex were added as covariates in subsequent analyses.

The current study adopted ethical principles outlined in the Declaration of Helsinki in 1964. Moreover, the Bioethics Committee of the institution where the study took place approved the study procedures.

### Measures

#### Sociodemographic information

Sociodemographic characteristics (e.g., age, biological sex, education) were queried with a self-report survey.

#### Alcohol use factors

The Short Inventory of Problems (54) was used to assess the maximum amount of alcohol consumed during consecutive heavy drinking periods, the number of consecutive days of heavy drinking, and the length of abstinence from alcohol use prior to the assessment through the use of a semi-structured interview. A modified version of the Substance Abuse Outcomes Module (55) was used to determine the duration of problematic alcohol use among individuals with AUD based on self-reported age of drinking problem onset.

#### Negative affect

The anxiety score from the Brief Symptoms Inventory [BSIanx; (56)] was used to assess negative affect severity. The BSI has been used as a valid indicator of negative affect and psychological distress (12). It was shown that negative affectivity may be more related to anxiety compared to depression (57). Cronbach’s α for the total BSI score was 0.97.

#### Alexithymia

The Polish version of the self-reported Toronto Alexithymia Scale [TAS-20; (58)] was used to assess alexithymia. Three subscale scores were assessed: (1) difficulty describing feelings (e.g., “It is difficult for me to find the right words for my feelings”; (2) difficulty identifying feelings (e.g., “I am often confused about what emotion I am feeling”; and (3) externally oriented thinking (e.g., “I prefer to analyze problems rather than describe them”; Cronbach’s αs = 0.60–0.84 across TAS subscales and total score). For the current study, a total score comprised of the sum of these subscales was analyzed.

#### Interoception

The modified version of Schandry’s heartbeat counting task [HCT; (59)] involves asking study participants to silently count their heartbeats across trials of different lengths (i.e., 25 s, 35 s and 45 s). The participants were told not to use helping strategies, such as assessing their pulse on their hand or neck. Actual heartbeats were recorded simultaneously using a standard electrocardiogram with 12 electrodes attached to the chest and limbs. The following formula was used to calculate interoceptive accuracy: 1/3∑(1 − (|actual heartbeats−reported heartbeats|)/actual heartbeats). A score of 1 equals a perfect match between self-reported and actual heartbeats. This method of measuring and calculating an *interoceptive accuracy index* is widely used in the field [e.g., (31, 39, 41, 60–62)].

### Data analysis

**Figure 1:**
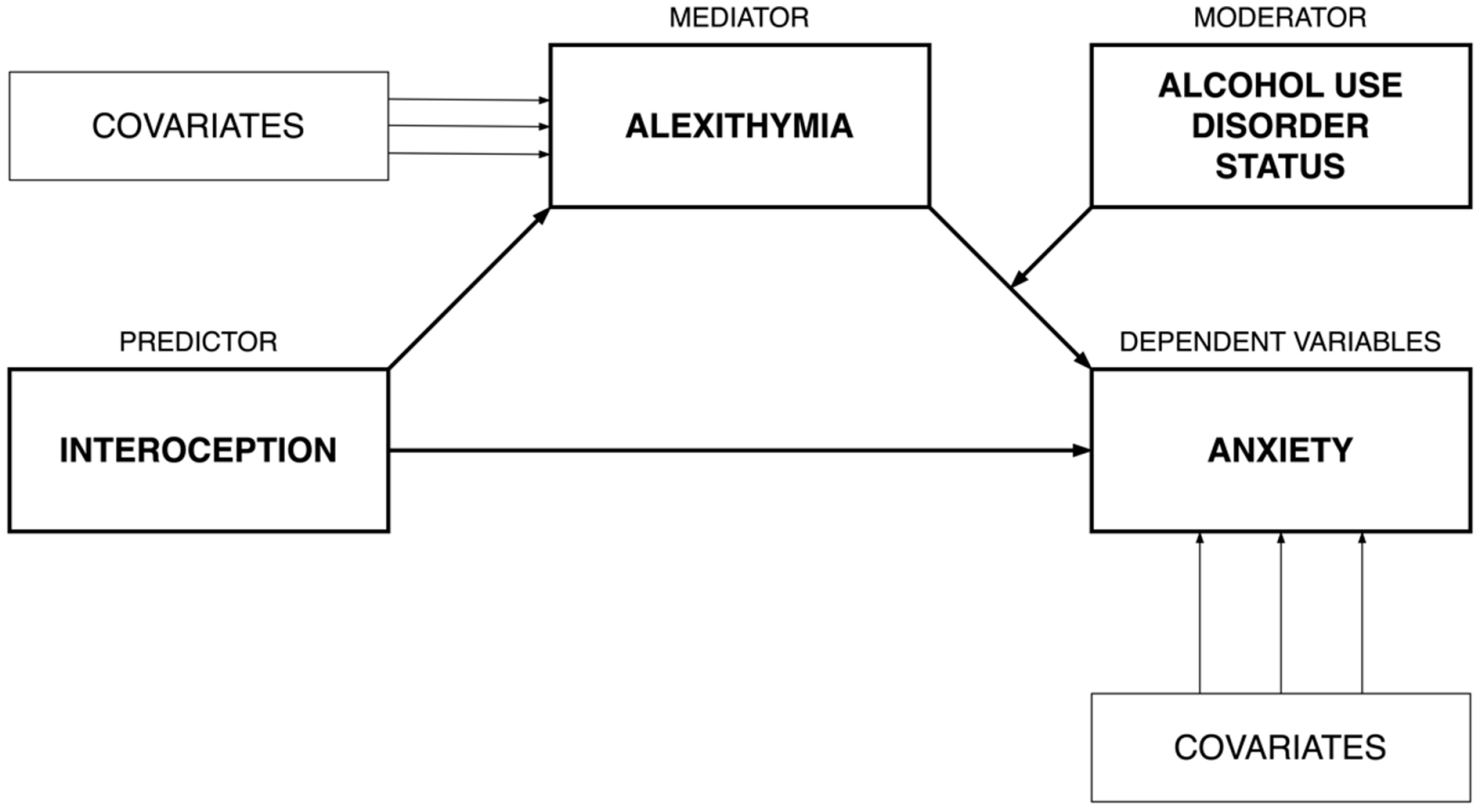
Conceptual diagram for moderated mediation model.

Means, standard deviations in interoceptive accuracy, alexithymia and anxiety severity in individuals with AUD and HCs are presented in Table 1. Correlations between interoceptive accuracy, alexithymia and anxiety severity in individuals with AUD and HCs are presented in Table 2. Hayes’ (63) PROCESS macro for SPSS to estimate moderated mediation with bootstrapping (5,000 resamples with replacement) was used to test AUD status as a moderator in the role of alexithymia as a mediator linking interoception and anxiety severity (see Figure 1 for a conceptual model). Namely, alexithymia was included as a mediator in the link between interoception on anxiety, with AUD status moderating the second association (i.e., the link between alexithymia and anxiety) while controlling for age and biological sex. Simple slope analyses were used to determine the nature of significant interactions. This entails estimating subsequent multiple regressions to assess the exact value of the moderator where the predictor (i.e., the mediator) has an effect on the dependent variable. Non-standardized coefficient values are presented. In PROCESS, an index of moderated mediation with a value outside of 0 is representative of an indirect effect that is conditional on the moderator (i.e., support for moderated mediation).

**Table 1:** Interoceptive accuracy, alexithymia, and anxiety severity in individuals with alcohol use disorder (AUD) and healthy controls (HC).

	AUD^a^ (*N* = 109^*^)	HC^a^ (*N* = 145^*^)	*t* ^b^	*p*
Interoceptive accuracy	0.72(0.08)	0.49(0.29)	7.74	< 0.001
TAS(total)	45.98(10.76)	56.51(11.21)	−7.53	< 0.001
TAS(ddf)	12.78(3.82)	15.01(3.86)	−4.59	< 0.001
TAS(dif)	15.08(4.49)	21.41(5.69)	−9.58	< 0.001
BSI(anx)	0.24(0.34)	0.88(0.76)	−8.21	< 0.001

Interoceptive accuracy – calculated using the following formula: 1/3∑(1 − (|actual heartbeats − reported heartbeats|)/actual heartbeats).

TAStotal – Toronto Alexithymia Scale total score; TASddf - Toronto Alexithymia Scale Difficulty Describing Feelings; TASdif - Toronto Alexithymia Scale Difficulty Identifying Feelings; BSIanx – Brief Symptom Inventory anxiety severity.

a Values are means and standard deviations.

b *t*-Student test was applied to measure the mean score difference between individuals with alcohol use disorder and healthy controls.

* for Schandry Test AUD N = 104 and HC *N* = 106.

**Table 2:** Correlations between interoceptive accuracy, alexithymia, and anxiety severity in individuals with alcohol use disorder (AUD) and healthy controls (HC).

		Interoceptive accuracy	TAS(total)	TAS(ddf)	TAS(dif)	BSI(anx)
Interoceptive accuracy	r (N)		−0.24^**^ (202)	−0.18^*^ (202)	−0.22^**^ (202)	−0.15^*^ (204)
TAS(total)	r (N)	−0.24^**^ (202)		0.79^**^ (254)	0.88^**^ (254)	0.39^**^ (253)
TAS(ddf)	r (N)	−0.18^*^ (202)	0.79^**^ (254)		0.65^**^ (254)	0.34^**^ (253)
TAS(dif)	r (N)	−0.22^**^ (202)	0.88^**^ (254)	0.65^**^ (254)		0.47^**^ (253)
BSI(anx)	r (N)	−0.15^*^ (204)	0.39^**^ (253)	0.34^**^ (253)	0.47^**^ (253)	

* Pearson two-sided correlation at the level < 0.05.

** Pearson two-sided correlation at the level < 0.01.

Interoceptive accuracy – calculated using the following formula: 1/3∑(1 − (|actual heartbeats − reported heartbeats|)/actual heartbeats).

TAStotal – Toronto Alexithymia Scale total score; TASddf - Toronto Alexithymia Scale Difficulty Describing Feelings; TASdif - Toronto Alexithymia Scale Difficulty Identifying Feelings; BSIanx – Brief Symptom Inventory anxiety severity.

## Results

**Figure 2:**
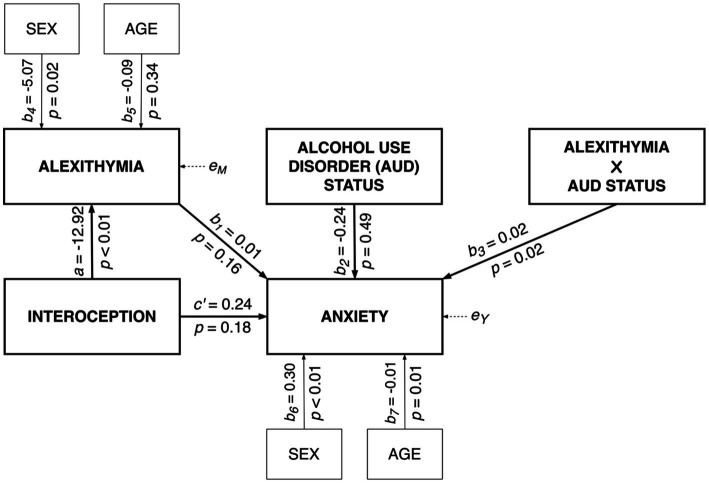
Moderated mediation model for anxiety.

**Figure 3:**
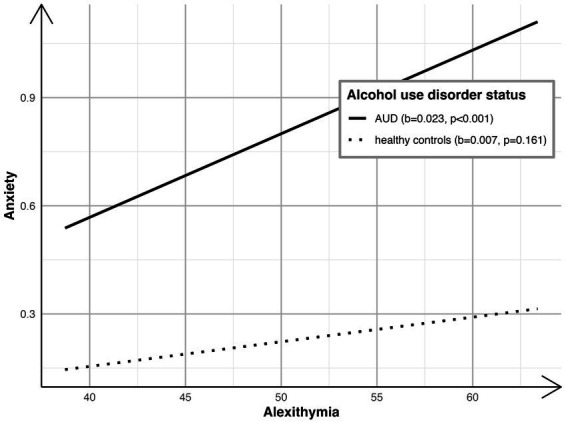
Alexithymia on anxiety by AUD status.

The moderated mediation model (63) with interoception (*HCT*) as a predictor, alexithymia (*TAS-20*) as mediator, and anxiety (*BSIanx*) as the dependent variable was tested (see Figure 2 for non-standardized coefficients). The model explained 9% of the variance in alexithymia (*R^2^* = 0.09; F[3,198] = 6.26; *p* < 0.01) and 38% of the variance in anxiety (*R^2^* = 0.38; F[6,195] = 20.10; *p* < 0.01). There was support for a significant two-way interaction between alexithymia and AUD status [*ΔR^2^* = 0.02; F(1,195) = 5.93; *p* = 0.02] on anxiety. As depicted in Figure 3, findings indicate that the simple slope for the regression of anxiety on alexithymia was statistically significant for individuals with AUD [*b* = 0.023; 95% CI = (0.014, 0.032); *p* < 0.001], but not for HCs (*b* = 0.007; 95% CI = [−0.003, 0.016]; *p* = 0.161). That is, alexithymia was positively associated with anxiety, but only among individuals with AUD. Nevertheless, the conditional indirect effect of interoception on anxiety via alexithymia was significant for individuals with AUD [*ab* = −0.300, bootstrap 95% CI = (−0.618, −0.088)], as well as HCs (*ab* = −0.088, bootstrap 95% CI = [−0.195, −0.014]). However, the conditional indirect effect across individuals with AUD and HCs differed significantly (index of moderated mediation: *ab_AUD_* - *ab_HC_* = −0.211, bootstrap 95% CI = [−0.497, −0.028]. That is, the role of alexithymia as a potential mediator in the association between interoception and anxiety was more pronounced for individuals with AUD compared to HCs and this may be due to a stronger link between alexithymia and anxiety among those with an AUD.

## Discussion

The main goal of this study was to investigate the interconnections between interoception, alexithymia, and negative affect (i.e., state anxiety) among individuals with AUD and a healthy control comparison group. To the best of our knowledge, this represents the first study testing whether alexithymia mediates the association between interoception (i.e., behaviorally measured cardiac interoceptive accuracy) and negative affect (i.e., current anxiety symptom severity), and if this association differs across groups (i.e., AUD vs. HC) using moderated mediation. The results show that across individuals with AUD and HCs, lower interoceptive accuracy is associated with greater alexithymia. In addition, AUD status was found to moderate the association between alexithymia and current anxiety symptom severity. Namely, alexithymia fully mediated the association between interoception and state anxiety in the same direction for both study groups, but the effect was significantly larger within the AUD sample.

It is commonly believed that alexithymia and interoception are related to each other. This is largely based on three factors: 1) according to some traditional theories of emotion, perception of bodily signals plays an important role in emotional processing (34); 2) alexithymia is present in a broad range of psychiatric disorders associated with decreased interoceptive abilities (64), and 3) certain brain regions engaged in interoceptive processing [e.g., the insula; (65)] are also associated with alexithymia (66). Modern theories even describe alexithymia as “a general deficit of interoception” (17). Nevertheless, despite the theoretical background, empirical research in this field is contradictory and methodologically inconsistent due to difficulties in conceptualizing and measuring interoception (47). For example, the authors of a recent meta-analysis found an association between self-reported interoception and alexithymia. Yet, the association between interoceptive accuracy and problems with identifying emotions (a component of alexithymia) was nonsignificant (47). In contrast, our study found evidence of a significant negative association between interoceptive accuracy and alexithymia across individuals with AUD and HCs. Individuals with impairments in recognizing interoceptive signals (i.e., heartbeat) reported greater alexithymia. Importantly, as stated earlier, it may be inappropriate to compare the results of studies utilizing subjective measures of interoception [see (47)] and those utilizing objective measures, as in the current study. Our findings are in line with earlier research, which confirmed a negative correlation between interoceptive accuracy and alexithymia in non-clinical samples (18, 39–41). However, as mentioned previously, there is some prior work supporting a positive correlation [e.g., (42)] or no correlation [e.g., (45)] between interoceptive accuracy and alexithymia. Thus, our results may supplement conflicting findings across the extant literature. Scarpazza and colleagues (61) put forward two hypotheses to explain mixed findings across the literature. The first suggests that individuals with alexithymia display impaired interoceptive abilities due to difficulties identifying and interpreting bodily changes on a cognitive level, which affects their subjective emotional experience. The other states that individuals with alexithymia may present heightened interoceptive accuracy because they experience emotions in a more “physical” way (42). These hypotheses are not mutually exclusive, as alexithymia may be the result of disturbed emotional processing at different levels of emotional awareness (36).

The association between interoception and alexithymia among individuals with AUD is understudied. This is surprising given the theoretical basis for such connections (26). Namely, deficits in the body’s sensing ability that contribute to difficulties in recognizing emotional states, which is characteristic of individuals with AUD (26), link alexithymia to interoception. Alexithymia was also found to moderate the association between subjective interoception and alcohol intake in individuals that binge drink (48). Moreover, neuroimaging studies showed numerous similarities between interoception, alexithymia, and neurobiological correlates of AUD [for review see (28)]. Consistent with prior work (32), we found that individuals with AUD demonstrating impaired interoceptive accuracy had greater alexithymia. However, Sönmez and colleagues (32) only found a negative correlation between interoceptive accuracy and a specific feature of alexithymia: difficulty identifying feelings in a sample of individuals with AUD. The authors of the latter study also indicated similar associations among individuals with varying substance use disorders, including heroin and synthetic cannabinoids (32). Our results may therefore expand upon the sparse empirical data to support an association between alexithymia, interoception, and problematic alcohol use.

As expected, our findings showed that high levels of alexithymia were associated with greater negative affect (i.e., current anxiety symptom severity) across individuals with AUD and a HC comparison group, but only significant in the former group. Prior work has supported associations between alexithymia and negative affect in HC samples (67), as well as in individuals with AUD (68). There is evidence that alexithymia is associated with anxiety. Research shows that difficulty recognizing emotions is related to both state anxiety and trait anxiety. Interestingly, among the different dimensions of alexithymia, two that are related to state anxiety are the inability to identify and describe feelings and to distinguish between feelings and bodily sensations (69, 70). Our findings indicate that alexithymia was significantly lower in the HC sample compared to individuals with AUD. It is plausible that only high levels of alexithymia may lead to increased levels of anxiety, while lower levels of alexithymia characterizing HCs may not be clinically relevant.

The main finding in this study that alexithymia mediated the association between interoception (interoceptive accuracy) and state anxiety may contribute to a greater understanding of the association between alexithymia, interoception, and alcohol misuse. According to several theories, bodily signals are important in the conscious experience of emotion. James and Lange (34) postulated that experiencing emotions was related to primary bodily changes. Damasio (35) later named the neural representations of these changes “somatic markers” that trigger emotions and drive behavior. More recent theories of embodiment claim that the mental representations of bodily changes formed during the original emotional experience are then reused when re-exposed to the emotional stimulus (71, 72). According to these theories, proper recognition of interoceptive signals is a prerequisite for consciously experiencing, distinguishing, and describing emotions. Thus, individuals high in alexithymia may experience anxiety due to abnormal interoception. Our results are consistent with this hypothesis and contribute to the expanding literature on the interoceptive basis of alexithymia (17, 18, 48).

Based on the above considerations, we believe that individuals with lower interoceptive abilities and associated difficulties in describing their emotional states are more likely to experience anxiety (i.e., unpleasant arousal). Alcohol may be used by these individuals as a coping strategy. This can lead to the development of addiction via negative reinforcement mechanisms. Interestingly, in individuals with AUD, as negative reinforcement increases, AUD severity also increases (73). This mechanism may be related to the negative effect of alcohol on interoceptive abilities and thus on abilities in describing one’s emotional states, which may in turn increase the level of aversive states, such as anxiety, fueling the vicious circle of addiction.

The results of our research can contribute to improving treatment programs for AUD and informing alcohol use prevention programs. Given that high alexithymia in individuals with AUD is associated with poorer treatment outcomes (74), improving emotion recognition may be an important therapeutic goal. A way to improve alexithymia may be to enhance interoceptive abilities. Previous data show that therapeutic approaches that target improving body awareness can increase one’s ability to recognize emotional states (75). More specifically, the work of Bornemann and Singer suggests that improvements in interoception affects the ability to recognize one’s own emotions, rather than vice versa (46).

This current study has some limitations. The main limitation of this study is the use of HCT to measure interoception. Despite the frequent use of this test in research on interoceptive accuracy, methodological weaknesses have been raised (18, 45, 76, 77). Namely, heartbeats may be perceived to some extent exteroceptively (77). The knowledge of the average heart rate may affect the results obtained using HCT (76). In their paper, Zamariola and colleagues summarized the main problems with HCT pointing out the following: 1) under-reporting of heartbeats; 2) low correlation between total actual heartbeats and total recorded heartbeats; 3) negative correlation between interoceptive accuracy and heart rate; and 4) differences in interoceptive accuracy scores across HCT trials (45). However, Ainley and colleagues commented on Zamariola’s paper and refuted most of the criticisms, they confirmed that HCT leads to underreporting of heartbeats, but that this limitation requires further empirical testing (78). Nevertheless, according to other research, the results of HCT correspond well with other interoceptive tasks (79). Recently, a study indicated that HCT can be a reliable test in assessing interoceptive accuracy (80). In the absence of alternative methods to study this phenomenon, we decided to use HCT, which will further allow us to compare our results with those obtained in prior work. However, we note the need for future research aimed at identifying additional methods to test interoceptive accuracy.

With regard to other limitations, the current study included participants from an inpatient treatment program for AUD with a severe course of the disorder and negative consequences of drinking. Thus, findings may not generalize to less severe cases of AUD. In addition, compared to HCs, individuals in the AUD group were older and more likely to be male. Although, both sex and age were used as control variables in all analyses, our results cannot be generalized to woman and/or individuals from other racial/ethnic groups. Older age in individuals with AUD may have also affected the results, as damage to the nervous system caused by long-term alcohol consumption may impair interoceptive accuracy. Moreover, due to the small number of women in the study, we cannot determine possible biological sex differences. We did not collect data on participants’ body weight in this study. This is a limitation as body weight may affect interoceptive accuracy (81).

In conclusion, the results of the current study show that alexithymia mediates the association between interoceptive impairment and negative affect. Moreover, the indirect effect was found to be significantly greater among individuals with AUD compared to HCs. Clinically, the current findings indicate that improving emotion recognition via therapeutic methods focused on strengthening interoceptive abilities could have utility for individuals receiving treatment for AUD. Future work empirically testing the value in bolstering interoception to enhance emotion recognition to buffer against negative affect among individuals with AUD should be conducted.

## Data availability statement

The raw data supporting the conclusions of this article will be made available by the authors, without undue reservation.

## Ethics statement

The studies involving humans were approved by Bioethics Committee of Medical University of Warsaw. The studies were conducted in accordance with the local legislation and institutional requirements. The participants provided their written informed consent to participate in this study.

## Author contributions

PW, AJ, ET, PK, HS, JZ, MR, and MK contributed to the conception and design of the work. PW, AJ, JZ, MR, and MK contributed to the acquisition of data. MK, ET, AJ, PK, and HS assisted with the analysis and interpretation of data. PW and MK managed the literature research and wrote the first draft of manuscript. AJ, ET, PK, HS, JZ, and MR revised and provided substantial input on the manuscript. All authors agreed to be accountable for all aspects of the work in ensuring that questions related to the accuracy or integrity of any part of the work are appropriately investigated and resolved. All authors contributed to the article and approved the submitted version.
